# eLife’s new model and its impact on science communication

**DOI:** 10.7554/eLife.84816

**Published:** 2022-12-08

**Authors:** Lara Urban, Mariana De Niz, Florencia Fernández-Chiappe, Hedyeh Ebrahimi, Laura KM Han, Devang Mehta, Regina Mencia, Divyansh Mittal, Elizabeth Ochola, Carolina Paz Quezada, Facundo Romani, Lana Sinapayen, Andy Tay, Aalok Varma, Lamis Yahia Mohamed Elkheir

**Affiliations:** 1 Helmholtz Pioneer Campus, Helmholtz AI, Technical University of Munich Munich Germany; 2 Institut Pasteur Paris France; 3 Instituto de Investigación en Biomedicina de Buenos Aires – CONICET – Partner Institute of the Max Planck Society, Buenos Aires, Argentina Buenos Aires Argentina; 4 Non-communicable Diseases Research Center, Tehran University of Medical Sciences Tehran Islamic Republic of Iran; 5 Centre for Youth Mental Health, University of Melbourne Parkville Australia; 6 Department of Biosystems, KU Leuven Leuven Belgium; 7 Instituto de Agrobiotecnología del Litoral (CONICET-UNL) Sante Fe Argentina; 8 Center for Integrative Genomics, Faculty of Biology and Medicine, University of Lausanne Lausanne Switzerland; 9 Centre for Global Health Research, Kenya Medical Research Institute Kisumu Kenya; 10 Departamento de Química Ambiental, Facultad de Ciencias, Universidad Católica de la Santísima Concepción Concepción Chile; 11 Department of Plant Sciences, University of Cambridge Cambridge United Kingdom; 12 Sony Computer Science Laboratories Kyoto Japan; 13 Department of Biomedical Engineering, National University of Singapore Singapore Singapore; 14 National Centre for Biological Sciences Bangalore India; 15 Department of Pharmaceutical Chemistry, Faculty of Pharmacy, University of Khartoum Khartoum Sudan

**Keywords:** peer review, preprints, scientific publishing, research assessment, early-career researchers, point of view, None

## Abstract

The eLife Early-Career Advisory Group discusses eLife’s new peer review and publishing model, and how the whole process of scientific communication could be improved for the benefit of early-career researchers and the entire scientific community.

## Introduction

Scientific progress relies on the rapid communication of trustworthy results within the scientific community. However, the current system for communicating new scientific results is slow, inefficient, and biased (Smith, 2006; Björk and Solomon, 2013; Helmer et al., 2017; Murray et al., 2019). One consequence of this slowness and inefficiency is that large sums of taxpayer money are wasted through inflated journal subscription costs and article processing charges (García et al., 2019).

Another problem is that many in the scientific community implicitly assume a strong correlation between the quality of a scientific article and the journal in which it was published. As a result, early-career researchers are encouraged to chase publications in certain journals (usually journals with high impact factors) in order to be competitive when it comes to securing fellowships, grants and jobs (Berenbaum, 2019). This view remains widespread despite efforts by many organizations to counter it – such as the San Francisco Declaration on Research Assessment (DORA, which stresses “the need to assess research on its own merits rather than on the basis of the journal in which the research is published”) and Plan S (which calls on funders to “value the intrinsic merit of the work and not consider the publication channel, its impact factor (or other journal metrics), or the publisher”). Individual funders – such as the European Molecular Biology Organization (EMBO), the Howard Hughes Medical Institute, the Max Planck Society and the Wellcome Trust – also support more sophisticated approaches to the assessment of science and individual scientists (Nicholas et al., 2018). We urge more organizations to do the same.

In recent years preprint servers such as bioRxiv and medRxiv have had a major impact on publishing in the life sciences and medicine because they allow scientists to publish their work when they feel it is ready, rather than having to wait for it to go through peer review and be published in a journal; indeed, it is now common for scientists to post a manuscript as a preprint at the same time as they submit it to a journal (Berg et al., 2016; Tennant, 2018; Puebla et al., 2021).

More recently various organizations have been leading efforts to promote the peer review of preprints. For example, OpenReview and PREreview are platforms that enable any scientist to review preprints, while Review Commons is an initiative led by EMBO that combines the public peer review of preprints with submission to a number of associated journals. In 2020, eLife announced that it would only review articles that were available as preprints (Eisen et al., 2020), and it created a platform called Sciety to aggregate public reviews of preprints from various sources.

All scientists are under pressure to publish, but early-career researchers face additional pressures when it comes to publishing. In particular, they are mostly on fixed-term contracts, so they are often preparing for their next career step, which requires them to rapidly demonstrate their productivity in their present position, most importantly through their publishing track record. However, it can take many months – or even years – for an article to be published under the current system, which seriously disadvantages early-career researchers (Sarabipour et al., 2019). While the advent of preprints means that it is now possible to demonstrate scientific productivity much sooner, the pressure of early-career researchers to publish in a small number of highly selective journals remains.

Journal titles and impact factors should never be used as proxies for research quality when making decisions about fellowships, grants and jobs.

We – as members of the eLife Early-Career Advisory Group – are strongly opposed to any form of research assessment that focuses on where a manuscript was published rather than on the merits of the research itself. We therefore believe that journal titles and impact factors should never be used as proxies for research quality when making decisions about fellowships, grants and jobs. We also deplore the emphasis on publishing in journals with high impact factors for two major reasons: first, journal impact factors only meaningfully evaluate journals, not individual manuscripts (Pendlebury, 2009); second, one’s chances of publishing in a high-impact-factor journal are strongly influenced by factors such as the host research group or institute and their available funding (Brown, 2007).

In this article, we discuss how the new eLife peer review and publishing model can affect the challenges that the current publishing system imposes on the scientific community in general, and on early-career researchers in particular. We also share our vision for the future of peer review and publishing in order to further increase efficiency, inclusion and equity, and to promote responsible behaviours in science.

## Advantages of the new eLife model

eLife recently announced that, from early 2023 onward, it would eliminate accept/reject decisions after peer review. Under this new model every manuscript that is sent for peer review will be published as a ‘reviewed preprint’ on the eLife website: this will include the preprint, public reviews by the eLife reviewers, and an eLife assessment written by the editor and reviewers (Eisen et al., 2022). This assessment will be based on a common vocabulary to provide a succinct, balanced assessment of the significance of the findings and the strength of the evidence reported in the manuscript. These eLife assessments can be used by readers to quickly contextualise manuscripts, and by authors to share rapidly in applications for fellowships, grants and jobs – thus avoiding many of the delays associated with traditional publishing (especially the time- and resource-intensive practice of requiring several rounds of revision and resubmission).

As scientific manuscripts are rarely universally perfect or universally flawed, the eLife assessments and public reviews will better reflect the details and nuance of the peer review process – both of which are lost when the peer review process is reduced to a binary accept/reject decision. While the new eLife model is therefore more transparent to readers, it also benefits authors who are incentivised to further improve their work while maintaining control over their own research program. The model therefore represents an important step towards giving all stakeholders an equal voice in the peer review and publishing process, and provides an opportunity to make the dialogue between authors, editors, and reviewers more fair and inclusive.

The new model will also allow negative results to be published, which will increase transparency in research and help protect early-career researchers and their mental health from the unpredictable nature of research as they seek to build a career (Mehta, 2019). The eLife assessment for a negative result might, for example, make clear that a manuscript scores highly on ‘strength of evidence’ even though the ‘significance of findings’ is limited, thus commending the authors for scientific rigor.

As scientific manuscripts are rarely universally perfect or universally flawed, the eLife assessments and public reviews will better reflect the details and nuance of the peer review process.

The new model marks a shift away from eLife as a journal publisher to eLife as an organization that peer-reviews preprints. In this model the author – rather than the editor and reviewers – decides when (if at all) to publish their reviewed preprint as a final Version of Record (in addition to deciding when to first publish the work as a preprint). Importantly, just as many funders have embraced preprints as evidence of scientific productivity, some are now also starting to embrace reviewed preprints. For example, EMBO now considers a first-author reviewed preprint as equivalent to a publication, which is one of the eligibility criteria for their postdoctoral fellowships (EMBO, 2022).

We believe that the new eLife model has the potential to make the scientific publishing system more just, equitable and inclusive. At present peer review by journals often disadvantages scientists, especially early-career researchers, from developing countries and other marginalised backgrounds (Desai, 2005; Murray et al., 2019). For instance, bias in peer review means that manuscripts from minority and women scientists are published less often in journals with high impact factors (Davies et al., 2021), and are also cited less often by other articles (Chatterjee and Werner, 2021). By making the assessment and evaluation of research more about the scientific content of an article, rather than the journal in which it is published, the new eLife model has the potential to give many more researchers access to fair, high-quality peer review. However, in order to make scientific publishing truly just, equitable and inclusive, it will be necessary for the population of scientists who perform gatekeeping roles for journals as editors and reviewers to be representative of the global population of scientists – and a lot of work is needed to achieve this goal.

## Overcoming persistent drawbacks

While we strongly support the new eLife model, we feel it retains some features of the current publishing system that remain problematic. First, the new model continues to rely on editorial triage, with a relatively small number of editors deciding which preprints are peer-reviewed and subsequently published as reviewed preprints by eLife. We therefore believe that eLife needs to establish mechanisms that will actively prevent any bias and inequity during this selection process. Decisions must be made in a way that is transparent and based on a clear set of rules in order to ensure that early-career researchers and minority scientists are not disadvantaged due to a lack of social networks or reputation.

In this context, eLife needs to increase its efforts to diversify its board of Senior and Reviewing Editors, and to make greater use of early-career researchers as both reviewers and editors (Mehta et al., 2020; eLife, 2020; eLife, 2021). We therefore encourage eLife to continue to expand its Early-Career Reviewer Pool (eLife, 2022a), and to run more open calls for Reviewing Editors, such as the recent call for Reviewing Editors from Latin America and the Caribbean (eLife, 2022b).

We are aware that many scientists, especially early-career researchers, are concerned that publicly receiving negative reviews and assessments for their preprints will somehow follow them through their careers and/or tarnish their reputation. However, the common vocabulary used in eLife assessments is intended to encourage editors and reviewers to provide constructive feedback. Thus, while committing to the new approach might seem risky, we are optimistic that the benefits far outweigh the risks. Moreover, eLife already has measures in place to mitigate these risks – such as consultative peer review, which results in more constructive feedback to authors.

The new eLife model has the potential to make peer review and the publication process faster, fairer, and more inclusive, rigorous and transparent.

We accept that high-quality peer review and the publication of reviewed preprints at eLife come at a cost (currently set at $2000), and we call on eLife to be as transparent as possible about the costs involved (subject to any legal restrictions on making such costs public), and to pledge that the cost charged will reflect the actual incurred cost. Waivers must also be available for those who cannot afford to pay, and clearly defined eligibility requirements for waivers should be made public. In order to further increase equity, we believe that – in the long term – the costs of peer review and publishing should be covered directly by funding agencies. We also feel that the scientific community as a whole further needs to rethink how reviewers can be adequately credited and compensated in the future. We strongly believe that working towards a more equitable and efficient peer review system should not be a solitary mission by eLife, but an effort by the entire scientific community including other journals and organizations.

It will also be important to build support for reviewed preprints in the scientific community, especially among funding agencies. While several funding agencies such as the Howard Hughes Medical Institute (HHMI, 2022a; HHMI, 2022b), the Wellcome Trust (Wellcome Trust, 2021) and EMBO (EMBO, 2022) already value reviewed preprints as evidence of scientific output, many others have not yet committed to do so. We therefore believe it important to create a bottom-up scientific movement that supports the new eLife approach to peer review and publishing. This movement should include funders, universities and research institutes from around the world, especially from systematically disadvantaged geographic locations such as in the Global South, and other organizations that have already altered the peer review and publishing system, such as Review Commons, DORA and ASAPbio. We also call on established scientists, including eLife editors, to demonstrate leadership by embracing the new approach for their own manuscripts and to lead the way from a position of privilege.

The new eLife model has the potential to make peer review and the publication process faster, fairer, and more inclusive, rigorous and transparent, and we as the eLife Early-Career Advisory Group wholeheartedly support it. We have identified several challenges and risks associated with the implementation of the new model, and we will therefore continue to make recommendations for future improvements to the editorial leadership of eLife. We strongly believe that such bold steps are needed to revolutionise research communication in order to ultimately build a more effective and inclusive research culture.

## Footnote

The authors are all past or present members of the eLife Early-Career Advisory Group (ECAG) and, as such, receive a small amount of financial compensation from eLife.

## References

[bib1] Berenbaum MR (2019). Impact factor impacts on early-career scientist careers. PNAS.

[bib2] Berg JM, Bhalla N, Bourne PE, Chalfie M, Drubin DG, Fraser JS, Greider CW, Hendricks M, Jones C, Kiley R, King S, Kirschner MW, Krumholz HM, Lehmann R, Leptin M, Pulverer B, Rosenzweig B, Spiro JE, Stebbins M, Strasser C, Swaminathan S, Turner P, Vale RD, VijayRaghavan K, Wolberger C (2016). Preprints for the life sciences. Science.

[bib3] Björk BC, Solomon D (2013). The publishing delay in scholarly peer-reviewed journals. Journal of Informetrics.

[bib4] Brown H (2007). How impact factors changed medical publishing - and science. BMJ.

[bib5] Chatterjee P, Werner RM (2021). Gender disparity in citations in high-impact journal articles. JAMA Network Open.

[bib6] Davies SW, Putnam HM, Ainsworth T, Baum JK, Bove CB, Crosby SC, Côté IM, Duplouy A, Fulweiler RW, Griffin AJ, Hanley TC, Hill T, Humanes A, Mangubhai S, Metaxas A, Parker LM, Rivera HE, Silbiger NJ, Smith NS, Spalding AK, Traylor-Knowles N, Weigel BL, Wright RM, Bates AE (2021). Promoting inclusive metrics of success and impact to dismantle a discriminatory reward system in science. PLOS Biology.

[bib7] Desai NG (2005). Why ′publish or perish′? Why not ′publish and prosper′? Perspectives from developing countries. Indian Journal of Psychiatry.

[bib8] Eisen MB, Akhmanova A, Behrens TE, Harper DM, Weigel D, Zaidi M (2020). Implementing a “publish, then review” model of publishing. eLife.

[bib9] Eisen MB, Akhmanova A, Behrens TE, Diedrichsen J, Harper DM, Iordanova MD, Weigel D, Zaidi M (2022). Peer review without gatekeeping. eLife.

[bib10] eLife (2020). The diversity of our editorial community. https://elifesciences.org/inside-elife/12096861.

[bib11] eLife (2021). Taking steps to increase the diversity of our editorial board. https://elifesciences.org/inside-elife/a25da8ff.

[bib12] eLife (2022a). Early-Careers Reviewer Pool: Authors can now select and nominate early-career reviewers for their work. https://elifesciences.org/inside-elife/eb42df87.

[bib13] eLife (2022b). Welcoming our newest editors in Latin America. https://elifesciences.org/inside-elife/bb4cc937.

[bib14] EMBO (2022). Refereed preprints in applications for EMBO postdoctoral fellowships. https://www.embo.org/features/refereed-preprints-in-applications-for-embo-postdoctoral-fellowships.

[bib15] García JA, Rodriguez-Sánchez R, Fdez-Valdivia J, Chamorro-Padial J (2019). The author’s ignorance on the publication fees is a source of power for publishers. Scientometrics.

[bib16] Helmer M, Schottdorf M, Neef A, Battaglia D (2017). Gender bias in scholarly peer review. eLife.

[bib17] HHMI (2022a). Open access to publications. https://hhmicdn.blob.core.windows.net/policies/Open-Access-To-Publications-Policy.

[bib18] HHMI (2022b). HHMI statement in support of eLife and open science innovation. https://www.hhmi.org/news/hhmi-statement-support-elife-and-open-science-innovation.

[bib19] Mehta D (2019). Highlight negative results to improve science. Nature.

[bib20] Mehta D, Bediako Y, de Winde CM, Ebrahimi H, Fernández-Chiappe F, Ilangovan V, Paz Quezada C, Riley JL, Saladi SM, Tay A, Weissgerber T (2020). Ways to increase equity, diversity and inclusion. eLife.

[bib21] Murray D, Siler K, Larivière V, Chan WM, Collings AM, Raymond J, Sugimoto CR (2019). Author-reviewer homophily in peer review. bioRxiv.

[bib22] Nicholas D, Herman E, Xu J, Boukacem-Zeghmouri C, Abdullah A, Watkinson A, Swigon M, Rodríguez-Bravo B (2018). Early career researchers’ quest for reputation in the digital age. Journal of Scholarly Publishing.

[bib23] Pendlebury DA (2009). The use and misuse of journal metrics and other citation indicators. Archivum Immunologiae et Therapiae Experimentalis.

[bib24] Puebla I, Polka J, Rieger OY (2021). Preprints: Their evolving role in science communication. MetaArXiv.

[bib25] Sarabipour S, Debat HJ, Emmott E, Burgess SJ, Schwessinger B, Hensel Z (2019). On the value of preprints: An early career researcher perspective. PLOS Biology.

[bib26] Smith R (2006). Peer review: A flawed process at the heart of science and journals. Journal of the Royal Society of Medicine.

[bib27] Tennant JP (2018). The state of the art in peer review. FEMS Microbiology Letters.

[bib28] Wellcome Trust (2021). Open Access Policy 2021 – Frequently Asked Questions. https://wellcome.org/sites/default/files/wellcome-open-access-policy-2021-faq.pdf.

